# In This Issue: Volume 116, Issue 10, October 2025

**DOI:** 10.1111/cas.70208

**Published:** 2025-10-01

## Nucleolar Organization in Response to Transcriptional Stress

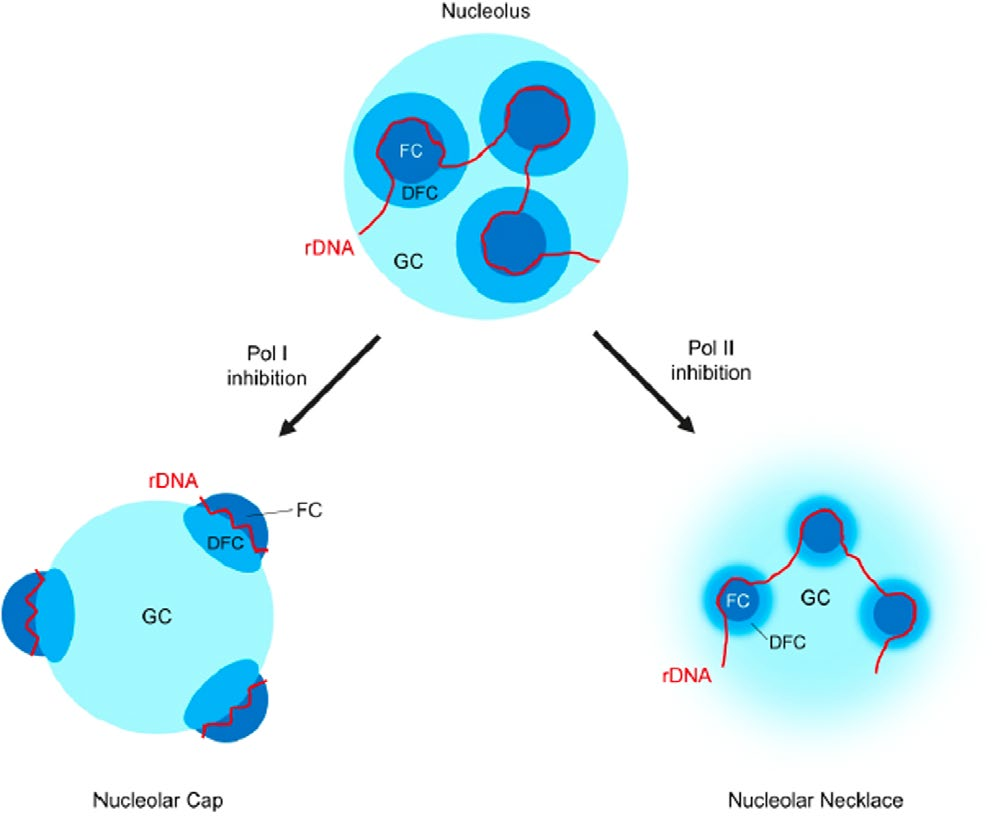

The nucleolus is a specialized structure within the nucleus that is best known for its role in ribosome production. Ribosomes are essential molecular machines that translate genetic information into proteins, making them critical for cell growth and survival. Ribosome biogenesis depends on stretches of DNA called rDNA, which are transcribed into ribosomal RNA before being processed and assembled into ribosomal subunits. Because this is a highly energy‐demanding process, the nucleolus is also highly sensitive to changes in the cellular environment.

In this issue, Imamura and Yasuhara review how the nucleolus responds to transcriptional stress and how these changes affect nucleolar organization. They describe the nucleolus as consisting of three distinct regions: one where rDNA is transcribed, one where the RNA undergoes processing, and one where ribosome components are assembled and exported. When RNA polymerase I‐ and/or RNA polymerase II‐dependent transcription is silenced, this three‐part structure is rearranged, and the nucleolus reorganizes into unusual structures known as nucleolar caps and necklaces.

Similar reorganizations occur when cells encounter stresses such as DNA damage within rDNA region, nutrient deprivation, or viral infection. Under these conditions, the nucleolus activates a protective mechanism called the nucleolar DNA damage response (n‐DDR). This response is not only structural but also functional, since the reorganized nucleolus recruits DNA repair enzymes that stabilize and protect rDNA from damage.

The authors note that genomic instability with an alternation in the rDNA copy number is frequently observed in cancer cells. This may occur due to impaired n‐DDR activity or reduced recruitment of repair proteins in the nucleolus. Chemotherapy drugs are also known to disrupt nucleolar function, underscoring the link between nucleolar reorganization and cancer treatment outcomes.

These findings highlight that the nucleolus is more than just a ribosome factory. It also functions as a central hub for genome maintenance and a critical sensor of cellular stress. A deeper understanding of n‐DDR could open the door to therapies that enhance DNA repair, reduce genomic instability, and improve outcomes for patients with cancer and other disorders linked to nucleolar stress.

https://onlinelibrary.wiley.com/doi/full/10.1111/cas.70164

## BCL‐2 Inhibitor Regulates Number, Function, and Antitumor Immunity of T Cells by Influencing Glycolysis in AML

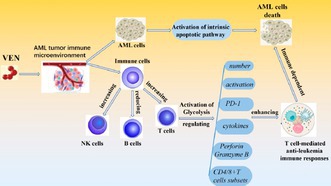

In this study, researchers investigating acute myeloid leukemia (AML), a form of blood cancer, discovered that the targeted drug Venetoclax uses the immune response to effectively kill leukemia cells and also enhances antitumor immune responses in AML.

AML cells often overexpress a protein called BCL‐2, which helps them avoid natural cell death. Venetoclax is a BCL‐2 inhibitor that induces programmed cell death in the cancer cells. While the direct effects of the drug on leukemia were known, its impact on the immune system had been less understood. This study explored how Venetoclax affects immune cells, particularly T cells, and the underlying biochemical pathways involved in its mechanism of action.

The researchers studied the effects of Venetoclax on AML cells and immune cells in vitro, from the cells obtained from the patients, and on the AML mice models. The researchers discovered that while Venetoclax effectively kills leukemia cells, it not only spares T cells from cell death, but it even boosts their cancer‐fighting abilities. This resistance is likely due to the T cells' low expression of BCL‐2. The drug also increased the activity and function of T cells by enhancing their production of molecules like IFN‐γ, Perforin, and Granzyme B, which help destroy cancer cells. It also increased the proportion of memory T cells, which are essential for long‐term immunity, and promoted T cell activation.

The underlying mechanism for this enhanced function appears to be that Venetoclax promotes a metabolic process called glycolysis in T cells. Glycolysis involves breaking down of glucose and fueling cell growth and providing them with the energy they need to fight the cancer.

These results show that molecular‐targeted drugs can have a positive impact on the immune system as well, opening the door for new combination therapies. The findings suggest that combining Venetoclax with immunotherapy or other targeted drugs could potentially improve outcomes for AML patients by leveraging both direct cancer cell killing and a strengthened immune response.

https://onlinelibrary.wiley.com/doi/full/10.1111/cas.70139

## Aurora‐A Promotes Cell‐Cycle Progression From Quiescence Through Primary Cilia Disassembly

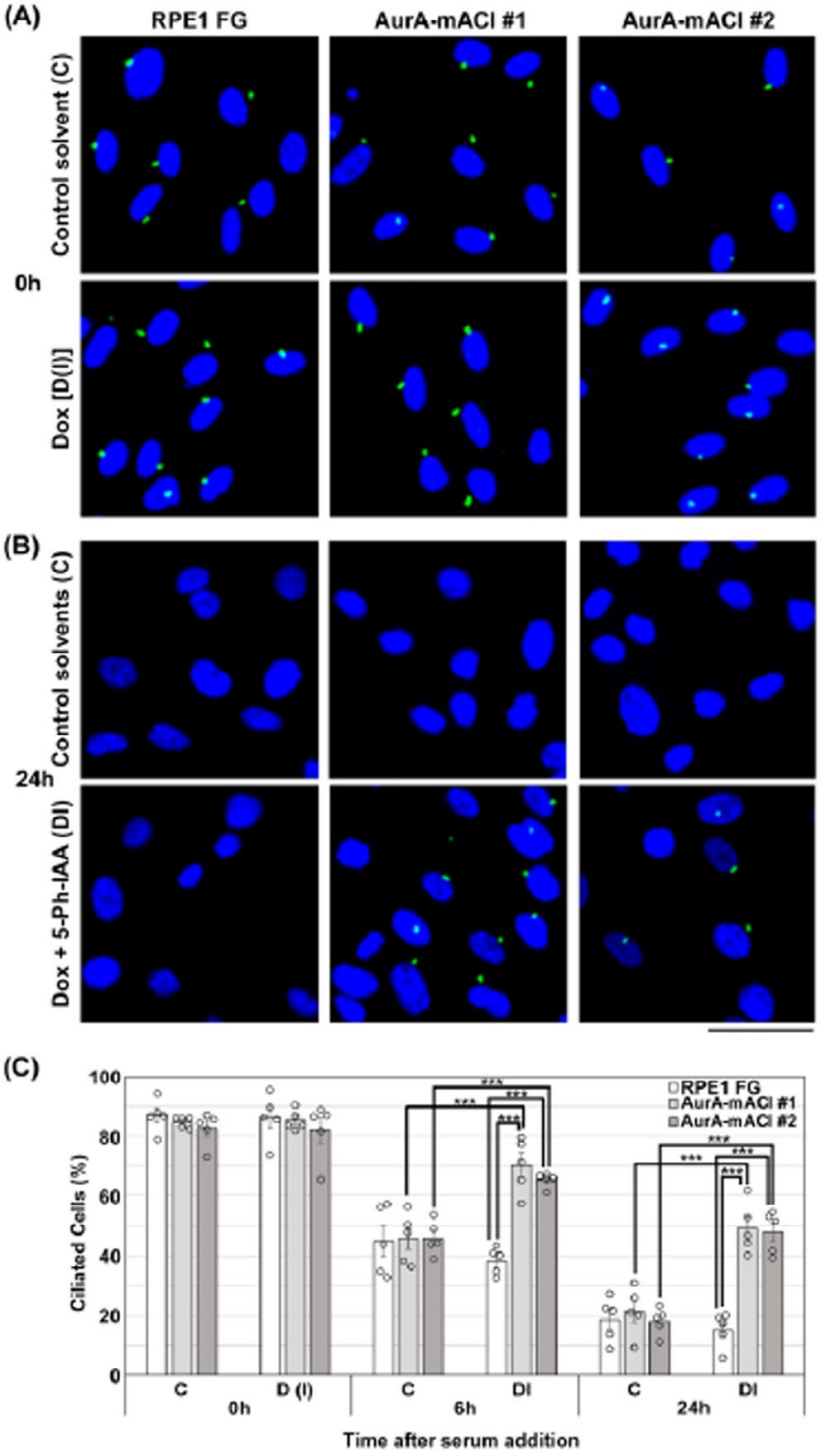

Aurora‐A (AurA) is a protein kinase best known for its role in mitosis, where it ensures that chromosomes are correctly distributed between daughter cells during division. Since cancer is characterized by uncontrolled cell division, AurA levels are often abnormally high in tumors, making it a major focus of drug development. However, less is known about what AurA does outside of mitosis, particularly in noncancerous cells.

In this study, researchers used CRISPR/Cas9 gene editing technology and a precise protein degradation system, auxin‐inducible degron 2 (AID2), to remove AurA from both noncancerous RPE1 cells and cancerous HCT116 cells. They discovered that in normal cells, AurA helps remove the primary cilia, which are tiny, hair‐like structures that form when cells are not actively dividing and act like cellular antennae. If they aren't removed, cells can't re‐enter the growth cycle efficiently.

When AurA was removed, the cilia didn't disassemble properly, causing delays in cell cycle re‐entry. However, when the researchers also removed IFT20, a protein required for cilia formation, the delay was reversed. This confirmed that the lingering cilia were responsible for the slowdown.

However, cancer cells like HCT116 cells typically lack primary cilia, and removing AurA not only slowed the cell cycle but also triggered cell death. When cilia formation was blocked in normal cells, they too became more sensitive to AurA loss, suggesting that cilia help protect normal cells under stress.

These findings suggest a double benefit for cancer therapy: inhibiting AurA kills cancer cells directly, while normal cells avoid death by entering a reversible rest state, thanks to their ability to form primary cilia.

https://onlinelibrary.wiley.com/doi/full/10.1111/cas.70153

